# Computer vision for evaluating retraction of the neurovascular bundle during nerve-sparing prostatectomy

**DOI:** 10.1007/s11701-025-02412-3

**Published:** 2025-06-02

**Authors:** Umar Ghaffar, Rikke Olsen, Atharva Deo, Cherine Yang, Jonathan Varghese, Randy G. Tsai, John Heard, Eman Dadashian, Carter Prentice, Peter Wager, Runzhuo Ma, Christian Wagner, Geoffrey A. Sonn, Alvin C. Goh, Graciela Gonzalez-Hernandez, Andrew J. Hung

**Affiliations:** 1 Department of Urology, Cedars Sinai Medical Center, Los Angeles, CA USA; 2 Department of Urology, Weill-Cornell Medical Center, New York, NY USA; 3 University of Southern California, Los Angeles, CA USA; 4 University of California, Los Angeles, David Geffen School of Medicine at UCLA, Los Angeles, CA USA; 5 Department of Urology and Urologic Oncology, St. Antonius-Hospital, Gronau, Germany; 6 Department of Urology, Stanford University Medical Center, Stanford, CA USA; 7 Department of Computational Biomedicine, Cedars Sinai Medical Center, Los Angeles, CA USA

**Keywords:** Retraction, Dissection gestures, Robotic surgery, Surgeon assessment, Prostatectomy

## Abstract

The nerve-sparing step of prostatectomy is crucial for post-operative sexual recovery, and excessive countertraction on the neurovascular bundle (NVB) during retraction has been associated with adverse sexual function outcomes. Our objective is to utilize computer vision to quantitatively assess the degree of this countertraction to study its impact on post-operative sexual recovery. Sixty-four nerve-sparing prostatectomy videos were used to extract snapshots prior to and at the maximum point of retraction gestures on the NVB. Semantic image segmentation, conducted with the Computer Vision Annotation Tool (CVAT), was used to label features such as the proportion of tissue grasped relative to retractor size and tissue stretch (measured by percent area increase and angular deviation from baseline). Supervised machine learning models, including Random Forest, Multi-layer Perceptron, and XGBoost, were then developed to predict the likelihood of erections sufficient for intercourse at a 12-month post-operative follow-up. Predictions were based on clinical and surgical gesture features (age, PSA, extent of nerve sparing, and post-operative Gleason scores, number of NVB retractions) alone and in combination with segmentation-derived features. One thousand one hundred four instances of NVB retraction were labeled. For patients with insufficient erectile function for intercourse at the 12-month follow-up, the mean angular deviation, percent area increase, and proportion of tissue grasped were 25.80° (SD 13.1), 41.81% (SD 33.3), and 0.310 (SD 0.093), respectively. In contrast, for patients with sufficient erectile function, these values were 21.07° (SD 7.4), 20.10% (SD 12.5), and 0.206 (SD 0.127), respectively. Integrating segmentation-derived features into the models enhanced predictive performance, with the AUC increasing from 0.78 (IQR 0.56–0.98) to 0.83 (IQR 0.63–1.00) for the Random Forest model, from 0.61 (IQR 0.35–0.85) to 0.74 (IQR 0.50–0.94) for the Multi-layer Perceptron, and from 0.70 (IQR 0.44–0.92) to 0.78 (IQR 0.58–0.97) for XGBoost. Delicate handling of the neurovascular bundle is crucial for better post-operative sexual recovery, and computer vision can provide an objective assessment of retraction on the NVB, offering insights beyond clinical and gesture features alone.

## Introduction

Preservation of the neurovascular bundle (NVB) during the nerve-sparing step of radical prostatectomy is pivotal for maintaining post-operative erectile function [1, 2]. Meticulous handling of tissue is essential to avoid neuropraxia or other injuries that may compromise erectile function [3]. Our previous work on quantifying surgical performance using surgical gestures has demonstrated that gestures can provide a granular method to objectively indicate surgical performance and outcomes [4]. Retraction, a supporting surgical gesture involving the manipulation of tissue to enhance visibility and access, plays a critical role in this process [5]. Hu et al. have also shown that techniques dissecting the prostate away from the NVB without countertraction, enable an earlier return of sexual function and potency [5].

Despite the recognized importance of gentle NVB handling, defining and quantifying what constitutes “excessive” retraction remains challenging. Traditional assessments often rely on a surgeon’s subjective judgment, which can vary significantly between operators [6, 7]. It is essential to establish objective metrics to assess surgical techniques and their influence on patient outcomes [4]. Advances in image-based assessment, including computer vision technologies, offer promising tools to provide more consistent and quantitative evaluations of surgical performance [8].

Our study addresses this critical limitation by introducing a comprehensive, quantitative approach to analyzing retraction on the neurovascular bundle (NVB). We focus on extracting key features from intra-operative images, including the amount of NVB tissue grasp, tissue stretch, and angular deviation from original position, and investigating their correlation with post-operative erectile function recovery. By bridging the gap in skill assessment, we aim to provide quantifiable evaluations of NVB retraction, ultimately offering surgeons objective feedback to optimize surgical technique and improve patient outcomes.

## Methods

### Data source

Sixty-four surgical videos of nerve-sparing robot-assisted radical prostatectomy performed at four institutions between 2016 and 2022 were analyzed. Videos were selected based on availability of corresponding clinical and follow-up data, including post-operative erectile function at 12 months. To reduce potential confounding, we included only patients with preserved baseline erectile function (Erection Sufficient for Intercourse or SHIM >17) and drew cases from high-volume academic centers where nerve-sparing was performed by experienced surgeons, minimizing variability due to comorbidities and surgical expertise. The nerve-sparing step was chosen as the key focus due to its critical impact on post-operative functional outcomes. Patients were stratified into two groups based on their 12-month post-operative erectile function: recovered group include patients who achieved erections sufficient for intercourse (ESI) (*n*=32) and non-recovered group include patients who did not recover sufficient erectile function (*n*=32). While we did not formally stratify surgical technique by center, all participating institutions used standard nerve-sparing robotic-assisted techniques. The majority of cases involved athermal dissection with selective clip usage, and a mix of antegrade and retrograde approaches was observed across the dataset.

### Gesture identification and annotation

Surgical gestures were identified and annotated by trained human annotators (UG, RM, JH, CY) through manual review. Using a predefined classification framework, gestures were categorized as various dissection or supporting gestures, including retraction as previously described. The annotation process involved watching the surgical videos and recording timestamps for specific gestures along with their anatomical location. Particular attention was given to instances of retraction on the NVB, which were marked for further analysis.

### Gesture utilization and skills assessment

Surgical gesture utilization and skills assessment were analyzed by comparing the total number of recorded gestures between groups with and without erections sufficient for intercourse at 12 months. Dissection Assessment for Robotic Technique (DART), a structured assessment tool used to evaluate surgical dissection skills in robotic-assisted procedures, scores were evaluated for tissue handling and tissue retraction to compare baseline dissection performance. Statistical comparisons were performed using appropriate tests to assess differences between groups, with significance set at *p* < 0.05.

### Feature extraction through image segmentation

**Fig. 1:**
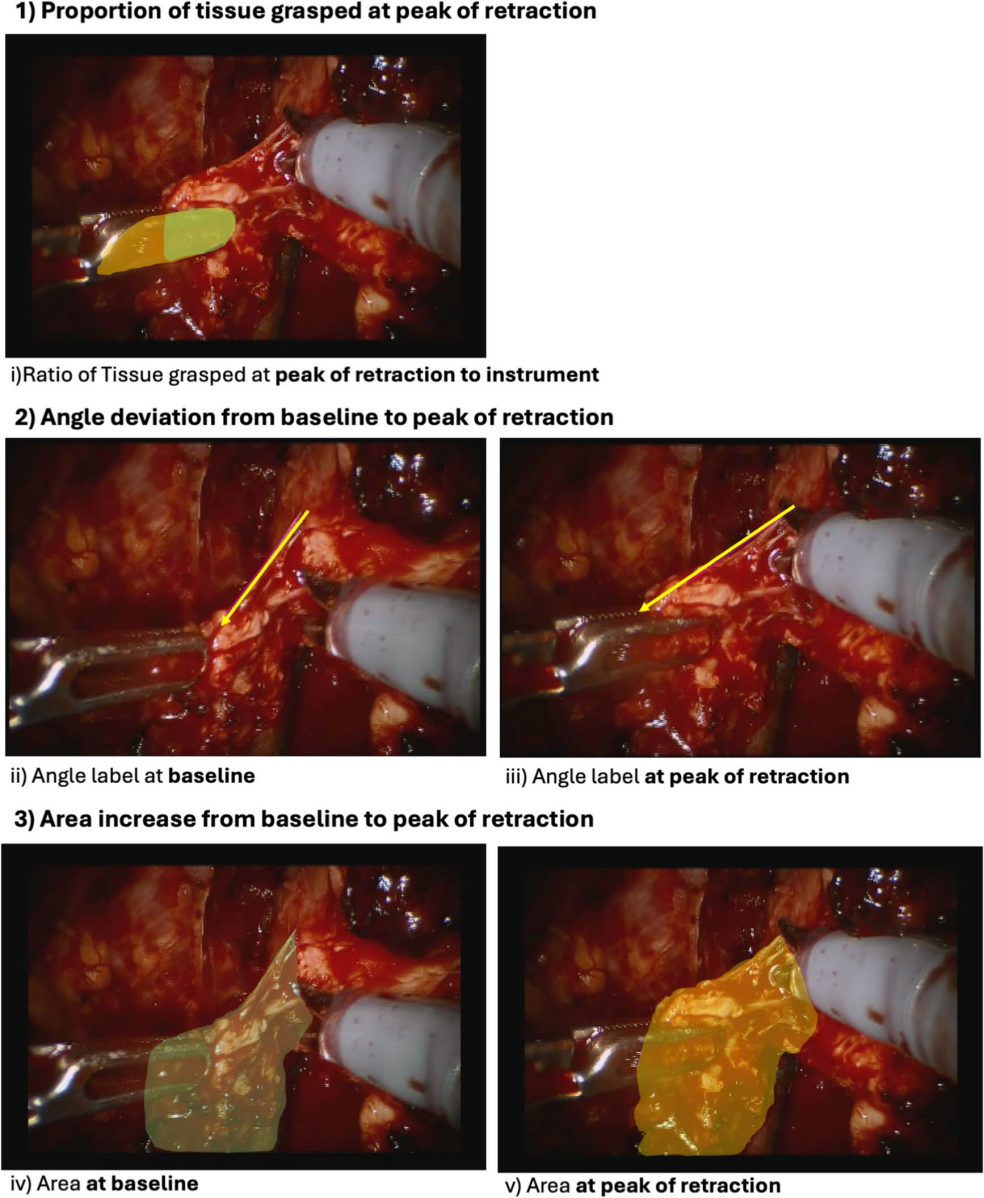
Images of retraction on neurovascular bundle (NVB) annotated for proportion of tissue grasped at peak of retraction; area and angle before and at peak of retraction on neurovascular bundle

For each instance of NVB retraction, we selected two specific frames for analysis: (1) the frame immediately prior to the initiation of retraction, representing the baseline anatomical state of the neurovascular bundle (NVB), and (2) the frame corresponding to the maximum visible retraction, defined as the point of peak tissue stretch and displacement as determined by the annotators during video review. These two frames were manually identified by annotators. Frames were excluded from segmentation if the retraction did not involve the NVB, if the retractor was not fully engaged with the tissue, or if the view of the NVB was obscured by surgical instruments, blood, or camera positioning. Semantic image segmentation was performed on these frames using the Computer Vision Annotation Tool (CVAT) (Fig. 1). This segmentation process involved manually labeling key features during active retraction of the NVB, including:

1. Proportion of tissue grasped: The ratio of the area of tissue held by the grasper to the overall grasper end-piece.
2. Percent area increase: The percentage change in NVB area between pre-retraction and maximum-retraction states, capturing tissue stretch.
3. Angular deviation: The change in NVB alignment relative to its baseline anatomical position.
4. Figure 1: Images of retraction on neurovascular bundle (NVB) annotated for proportion of tissue grasped at peak of retraction; area and angle before and at peak of retraction on neurovascular bundle.

### Inter-rater reliability

These features were extracted directly from the segmented images and validated through consensus among annotators to ensure consistency and reliability. Inter-rater reliability for segmentation-derived features including percentage area increase, angular deviation and proportion of tissue grasped was calculated among the raters using intra-class correlation coefficient (ICC).

### Statistical analysis and machine learning models

Several supervised machine learning models including Random Forest (RF), Multi-layer Perceptron (MLP), and XGBoost were trained to assess the impact of clinical and gesture features alone versus a combination of image-based, clinical, and gesture features on post-operative erectile function recovery.

In our analysis, the dataset of 64 cases was randomly split into a training set (70%, *n*=45) and a test set (30%, *n*=19) while preserving the outcome distribution (i.e., balanced proportion of recovered and non-recovered erectile function cases) to avoid class imbalance. The training set was used for model development and hyperparameter tuning via cross-validation, while the test set was held out entirely for final performance evaluation. The performance of these models was evaluated using the area under the receiver operating characteristic curve (AUC). Statistical tests, including t tests and Chi-square analyses, were employed to compare retraction features between outcome groups and identify significant trends. To mitigate overfitting risks from the small dataset and multiple models, we used stratified fivefold cross-validation and nested cross-validation for hyperparameter tuning. Regularization (L2, dropout for MLP; early stopping and regularization for XGBoost) was applied, and model complexity was limited by selecting clinically and statistically relevant features. Performance metrics were reported with confidence intervals to reflect variability and uncertainty.

Two feature configurations were used for model training and evaluation:

1. Clinical and gesture features: Included patient age, PSA levels, extent of nerve sparing (surgeon reported), post-operative Gleason scores, and the number of NVB retractions (gesture count).
2. Segmentation features included with clinical and gesture features: Integrated clinical and gesture features with image-based metrics derived from segmentation, including the proportion of tissue grasped, percent area increase, and angular deviation.

## Results

### Dataset overview

The dataset consisted of 64 cases of nerve-sparing prostatectomy. Patients were stratified based on post-operative erectile function recovery at the 12-month follow-up. The median number of surgical gestures per case was 630 (IQR 433–980) in the group with erections sufficient for intercourse at 12 months and 521 (IQR 292–661) in the group without sufficient erections (Table 1). The median number of retraction gestures per case was 75 (IQR 48–126) in recovered and 68 (IQR 38–79) in non-recovered groups. The median number of retractions on the NVB per case was 12 (IQR 4–19) in recovered and 15 (IQR 9–21) in non-recovered groups. Mean scores for skills assessment via Dissection Assessment for Robotic Technique (DART) were not significantly different for tissue handling (*p* = 0.794) and tissue retraction (*p* = 0.379) domains among the two group.

**Table 1:** Baseline characteristics of recovered vs non-recovered erectile function groups at 12-month post-operatively

Variable	Erectile function recovery at 12 months (*n* = 32)	No erectile function recovery at 12 months (*n* = 32)
Clinical features		
Age (mean, SD)	60.7 (6.5)	65.7 (7.1)
PSA—preoperative ng/ml (Mean, SD)	7.8 (3.8)	8.1 (4.9)
Extent of nerve spare		
Full	30/32	27/32
Partial	2/32	5/32
Extra-capsular extension (T3a or above)	17/32	20/32
Gleason—post-operative		
≤ 7	30/32	26/32
Surgical gesture utilization		
Gestures per case (median-IQR)	630 (433–980)	521 (292–661)
Retraction per case (median-IQR)	75 (48–126)	68 (38–79)
Retractions on NVB per case (median-IQR)	12 (4–19)	15 (9–21)
Skills assessment by Dissection Assessment for Robotic Technique (DART)		
Instrument visualization (median-IQR)	2.88 (2.80–3)	3 (2.80–3)
Respect for tissue plane (median-IQR)	2.87 (2.76–3)	2.94 (2.69–3)
Tissue handling (median-IQR)	2.61 (2.30–2.79)	2.56 (2.19–2.70)
Tissue retraction (median-IQR)	2.69 (2.50–2.92)	2.83 (2.50–3)
Efficiency (median-IQR)	2.58 (2.40–2.77)	2.70 (2.38–2.84)

### Quantitative features extracted for retraction on NVB

One thousand one hundred four instances of retractions on the NVB were segmented using CVAT and the following features were derived.

**Fig. 2:**
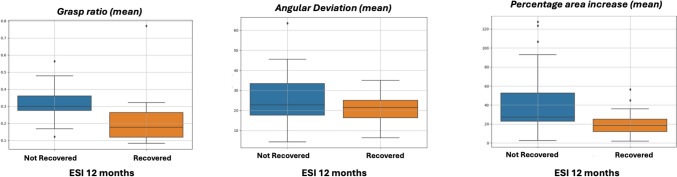
Box plot illustrating mean grasp ratio, angular deviation, and percentage area increase in groups that did not recover versus recovered erectile function

1. Angular Deviation of NVBThe angular deviation of the neurovascular bundle during retraction was lower in patients who recovered erectile function with an average deviation of 21.07° (SD 7.4), compared to 25.80° (SD 13.1) in non-recovered cases. (Fig. 2)
2. Percentage NVB Area IncreaseRecovered cases exhibited a lower percentage area increase, representing reduced NVB stretch during maximum retraction. The mean area increase was 20.10% (SD 12.5) in recovered patients, compared to 41.81% (SD 33.3) in non-recovered patients (Fig. 2).
3. Proportion of NVB Tissue GraspedThe proportion of NVB tissue grasped relative to the grasper end-piece size was significantly lower in recovered cases with a mean tissue grasp of 0.206 (SD 0.127), compared to 0.310 (SD 0.093) in non-recovered patients (Fig. 2).

### Inter-rater reliability

Inter-rater reliability assessed using intraclass correlation coefficient (ICC) between two raters trained to do image segmentation on 10 training cases was 0.74 (SD 0.04) for angular deviation, 0.76 (SD 0.08) for proportion of tissue grasped, and 0.68 (SD 0.07) for percentage area increase.

### Predictive modeling

Machine learning models were employed to predict post-operative erectile function recovery using clinical and gesture features in the first experiment and these features in combination with image-based retraction metrics in the second experiment. Model performance was evaluated using metrics such as the area under the curve (AUC), accuracy, precision, recall, and F1 score (Table 2).

**Fig. 3:**
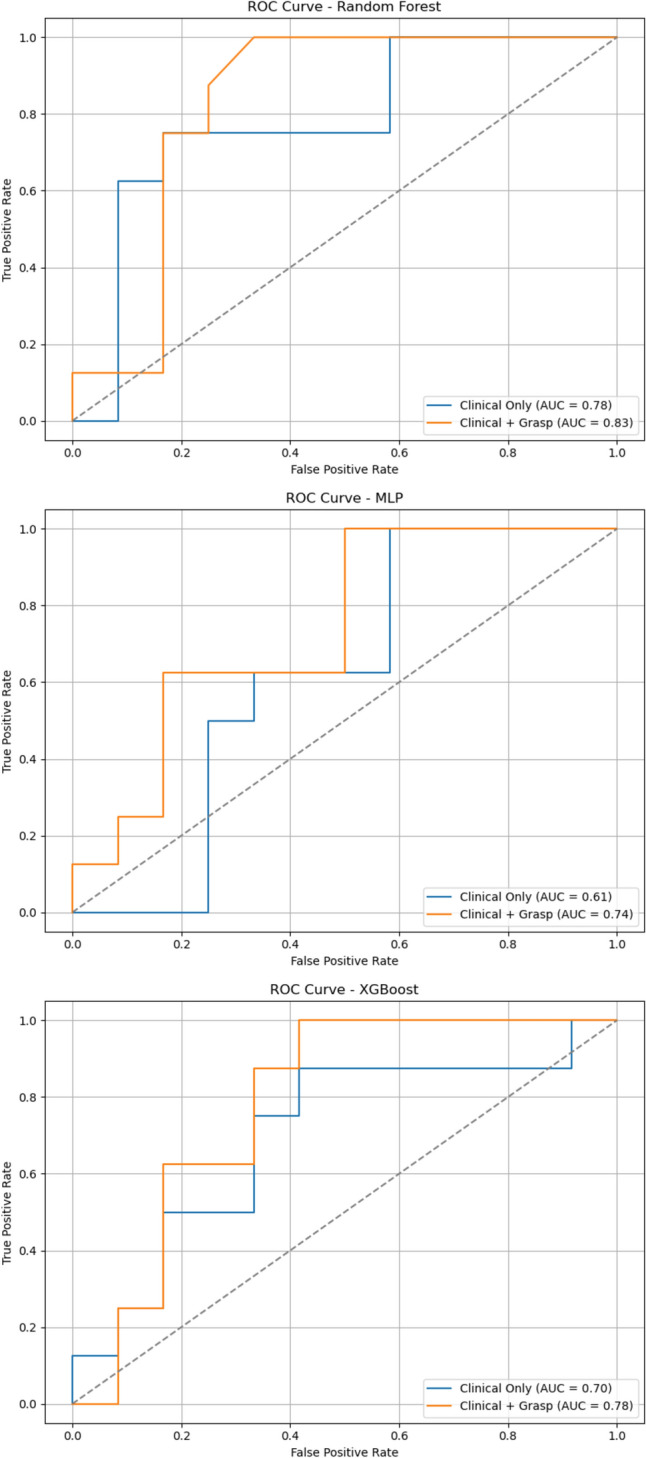
Receiver Operating Characteristic (ROC) for random forest, multi-layer perceptron and XGBoost classifiers for predicting erectile function at 12 months post-operatively

1. Clinical and Gesture Features OnlyWhen trained solely on clinical and gesture features (age, PSA, nerve-sparing extent, post-operative Gleason score, and the number of NVB retractions), the Random Forest (RF) model achieved an AUC of 0.78 (95% CI: 0.56–0.98), with an accuracy of 70% and an F1 score of 0.67. The MLP and XGBoost models performed less favorably, with AUCs of 0.61 (95% CI: 0.35 – 0.85) and 0.70 (0.44 – 0.92), respectively, and lower precision and recall values (Fig. 3).
2. Combined Clinical and Image-Based FeaturesIncorporating image-based features (proportion of tissue grasped, percentage area increases, and angular deviation) alongside clinical variables significantly improved predictive performance across all models:
  o Random Forest: AUC increased to 0.83 (95% CI: 0.63–1.00), with improved accuracy (75%) and F1 score (0.76).
  o MLP: AUC increased to 0.74 (95% CI: 0.50–0.94), with accuracy of 65% and recall of 0.88.
  o XGBoost: AUC improved to 0.78 (95% CI: 0.58–0.97), with accuracy of 65% and a precision of 0.56.

**Table 2:** Performance metrics of Random Forest, Multi-layer Perceptron, and XGBoost classifiers for predicting erectile function at 12 month post-operatively

Clinical and gesture features only			
Metric	Random Forest	MLP	XGBoost
AUC	0.78 (0.56–0.98)	0.61 (0.35–0.85)	0.70 (0.44–0.92)
Accuracy	0.70	0.60	0.65
Precision	0.60	0.50	0.54
Recall	0.75	0.63	0.75
F1 score	0.67	0.56	0.63

Clinical and gesture along with image segmentation features			
Metric	Random Forest	MLP	XGBoost
AUC	0.83 (0.63–1.00)	0.74 (0.50–0.94)	0.78 (0.58–0.97)
Accuracy	0.75	0.65	0.65
Precision	0.62	0.54	0.56
Recall	1.00	0.88	0.63
F1 score	0.76	0.67	0.59

In summary, patients with recovered erectile function exhibited less angular deviation, less tissue stretch, and a less proportion of tissue grasped during NVB retraction. Integrating image-based metrics with clinical and gesture features significantly improved the predictive performance of machine learning models, particularly Random Forest, which achieved the highest AUC (0.83).

## Discussion

Our study demonstrates that excessive neurovascular bundle (NVB) retraction, as assessed through quantitative features extracted from intra-operative images such as angular deviation, percentage area increases, and proportion of tissue grasped, is associated with worse post-operative erectile function outcomes, suggesting that excessive tissue handling during nerve-sparing prostatectomy critically influences functional recovery. By incorporating features extracted from image analysis and machine learning (ML) techniques, we were able to enhance the prediction of erectile function recovery beyond the use of clinical and gesture features alone.

Innovations in nerve-sparing technique during radical prostatectomy have been pivotal in improving post-operative erectile function outcomes. Factors affecting these reported outcomes range from surgical technique, methods of data collection (patient vs physician-reported), and patient selection. Hu et. al have described improved 5-month sexual function outcomes in their nerve-sparing surgical technique without active NVB countertraction by the assistant or surgeon [5]. Similarly, Mulhall et al. have identified NVB countertraction as a source of postprostatectomy neurogenic erectile dysfunction (ED) [9]. While it is established that manipulation of the neurovascular bundle may damage the nerve resulting in neuropraxia and post-operative sexual dysfunction, it is unknown if the extent of this retraction determines outcomes. These studies highlight the critical role of controlled surgical maneuvers and their direct impact on functional outcomes, further advocating for the development of objective metrics to evaluate and train surgeons in these techniques.

In our previous work where we decoded the nerve-sparing step into its fundamental surgical gestures, we found that excessive frequency of grabs on the neurovascular bundle had independent association with worse operative erectile outcomes as assessed by the *Erection Sufficient for Intercourse* component of Sexual Health Inventory for Men (SHIM) at 12 months. However, frequency of NVB grasps alone does not capture the full scope of NVB manipulation. This study extends that foundation by assessing the extent of NVB retraction through detailed intra-operative video analysis. By quantifying retraction metrics, such as tissue stretch and angular deviation, we elucidate the direct impact of retraction severity on post-operative erectile function recovery enabling a more comprehensive understanding of how surgical techniques influence NVB integrity and patient outcomes.

We found that increased relative area increase, angular deviation, and proportion of tissue grasped correlate strongly with poorer erectile outcomes. These metrics serve as proxies for tissue stretch and distortion, providing insight into the degree of mechanical stress experienced by the NVB during surgery. While the absolute differences in retraction metrics (e.g., angular deviation) were modest, their value lies in reflecting consistent behavioral patterns during nerve-sparing dissection. These patterns may represent subtle yet reproducible variations in surgical technique that, over time, contribute to differential NVB outcomes. As such, the findings highlight potential markers of tissue handling quality rather than fixed clinical thresholds. By combining features such as angular deviation, tissue stretch, and proportion of tissue grasped with clinical variables like patient age and extent of nerve sparing, our ML models outperformed traditional approaches relying on clinical data alone. For instance, the incorporation of these quantitative metrics significantly improved model performance metrics. These findings suggest that the observed correlations are likely attributed to stretch-induced neuropraxia and associated mechanical trauma to the NVB [3]. While our results demonstrate a strong association between quantitative NVB retraction features and erectile function outcomes, we acknowledge that this is an observational study and does not establish causality. The relationship between mechanical manipulation and neuropraxia requires further investigation through prospective or experimental studies.

We envision several impactful clinical applications of this work. The integration of machine learning (ML)-based image analysis into the surgical console or robotic system could enable real-time surgeon guidance during critical steps such as nerve sparing. By continuously analyzing intra-operative video feeds, the system could provide automated alerts or visual cues when retraction metrics such as tissue stretch, angular deviation, or proportion of tissue grasped exceed predefined safety thresholds, thereby helping to prevent potential nerve damage. This would function similarly to a “retraction warning system,” enhancing intra-operative awareness without disrupting workflow. The quantitative metrics developed through our segmentation approach could serve as objective performance feedback tools and could be incorporated into post-operative debriefs surgical training programs.

This study has several limitations that warrant discussion and provide avenues for future improvement. First, the reliance on a single-camera setup without depth perception introduces challenges in accurately quantifying three-dimensional NVB retraction metrics. The lack of depth information restricts the assessment to two-dimensional changes, such as relative area increase and angular deviation, potentially underestimating the true extent of tissue manipulation. Future studies should consider incorporating 3D modalities, which could provide precise volumetric assessments of NVB deformation and improve the fidelity of the metrics. In addition, the manual annotation process, though validated through inter-rater reliability measures, remains time-intensive and prone to variability. The moderate inter-rater reliability values (ICC range: 0.68–0.76) for segmentation-derived features highlight the inherent variability in manual image annotation from intra-operative video. To address this, we employed a consensus review process among trained annotators to enhance consistency. Future work will focus on developing automated segmentation pipelines using deep learning to improve reproducibility and efficiency. Automation of the annotation process using advanced deep learning frameworks, such as convolutional neural networks (CNNs) trained for surgical gesture recognition and segmentation, could significantly enhance the scalability and consistency of the methodology. Automating these steps would not only reduce observer bias but also enable real-time feedback during surgery, paving the way for intra-operative guidance systems. A limitation of our study is the use of the 12-month post-operative time point to assess erectile function recovery, as data beyond this period were not uniformly available across the cohort, potentially underestimating longer term recovery outcomes. A key limitation of this study is the relatively small sample size (64 cases), which may constrain the generalizability and robustness of our machine learning models. While the present study serves as a proof-of-concept for the integration of computer vision-based retraction metrics into surgical outcome prediction, we acknowledge that larger datasets are essential for more reliable model training and validation. Ongoing prospective efforts across multiple institutions are currently underway to expand our dataset, which will enable more robust model development. An important limitation of this study is the lack of external validation. While we utilized an internal train-test split to evaluate model performance, the absence of an independent validation cohort limits the generalizability of our findings.

## Conclusion

This study highlights the critical importance of quantifying NVB retraction during nerve-sparing prostatectomy, demonstrating its direct correlation with erectile function outcomes. By integrating computer vision-derived metrics with clinical data, we provide a robust framework for evaluating and improving surgical performance. Future directions include the expansion of datasets, incorporation of long-term functional recovery metrics, and development of real-time feedback systems. Such advancements will pave the way for objective surgeon skill assessment and targeted training interventions, ultimately improving patient outcomes.

## Data Availability

The data that support the findings of this study will be made available to the public as open access at https://zenodo.org/communities/uscurologycrse/records?q=&l=list&p=1&s=10&sort=newest. However, this will not be completed until the completion of data collection and de-identification from all sites.

## References

[1] Carter S, Le JD, Hu JC (2013) Anatomic and technical considerations for optimizing recovery of sexual function during robotic-assisted radical prostatectomy. Curr Opin Urol. 23(1):88–94. 10.1097/MOU.0b013e32835b6602 23169152 10.1097/MOU.0b013e32835b6602

[2] Tewari AK, Srivastava A, Huang MW, Robinson BD, Shevchuk MM, Durand M et al (2011) Anatomical grades of nerve sparing: a risk-stratified approach to neural-hammock sparing during robot-assisted radical prostatectomy (RARP). BJU Int. 108(6 Pt 2):984–92. 10.1111/j.1464-410X.2011.10565.x 21917101 10.1111/j.1464-410X.2011.10565.x

[3] Basourakos SP, Kowalczyk K, Moschovas MC, Dudley V, Hung AJ, Shoag JE et al (2021) Robot-assisted radical prostatectomy maneuvers to attenuate erectile dysfunction: technical description and video compilation. J Endourol. 35(11):1601–9. 10.1089/end.2021.0081 34015959 10.1089/end.2021.0081 PMC8820193

[4] Ma R, Ramaswamy A, Xu J, Trinh L, Kiyasseh D, Chu TN et al (2022) Surgical gestures as a method to quantify surgical performance and predict patient outcomes. NPJ Digit Med. 5(1):187. 10.1038/s41746-022-00738-y 36550203 10.1038/s41746-022-00738-y PMC9780308

[5] Kowalczyk KJ, Huang AC, Hevelone ND, Lipsitz SR, Yu HY, Ulmer WD et al (2011) Stepwise approach for nerve sparing without countertraction during robot-assisted radical prostatectomy: technique and outcomes. Eur Urol. 60(3):536–47. 10.1016/j.eururo.2011.05.001 21620561 10.1016/j.eururo.2011.05.001

[6] Zhang S, Cui XM, Du T, Ma CY, Hu RH, Yuan B et al (2023) Application of a new retraction method in laparoscopic gastrectomy for gastric cancer. Surg Laparosc Endosc Percutan Tech. 33(4):431–4. 10.1097/sle.0000000000001193 37311036 10.1097/SLE.0000000000001193

[7] Steele PR, Curran JF, Mountain RE (2013) Current and future practices in surgical retraction. Surgeon. 11(6):330–7. 10.1016/j.surge.2013.06.004 23932799 10.1016/j.surge.2013.06.004

[8] Baghdadi A, Hussein AA, Ahmed Y, Cavuoto LA, Guru KA (2019) A computer vision technique for automated assessment of surgical performance using surgeons’ console-feed videos. Int J Comput Assist Radiol Surg. 14(4):697–707. 10.1007/s11548-018-1881-9 30460490 10.1007/s11548-018-1881-9

[9] Mulhall JP, Bella AJ, Briganti A, McCullough A, Brock G (2010) Erectile function rehabilitation in the radical prostatectomy patient. J Sex Med. 7(4 Pt 2):1687–98. 10.1111/j.1743-6109.2010.01804.x 20388165 10.1111/j.1743-6109.2010.01804.x

